# Design and Evaluation of a Capacitive Micromachined Ultrasonic Transducer(CMUT) Linear Array System for Thickness Measurement of Marine Structures Under Varying Environmental Conditions

**DOI:** 10.3390/mi16080898

**Published:** 2025-07-31

**Authors:** Changde He, Mengke Luo, Hanchi Chai, Hongliang Wang, Guojun Zhang, Renxin Wang, Jiangong Cui, Yuhua Yang, Wendong Zhang, Licheng Jia

**Affiliations:** State Key Laboratory of Extreme Environment Optoelectionic Dynamic Measurement Technology and Instrument, North University of China, Taiyuan 030051, China; hechangde@nuc.edu.cn (C.H.); s202306010@st.nuc.edu.cn (M.L.); s202306026@st.nuc.edu.cn (H.C.); wanghongliang@nuc.edu.cn (H.W.); zhangguojun1977@nuc.edu.cn (G.Z.); wangrenxin@nuc.edu.cn (R.W.); jgcui@nuc.edu.cn (J.C.); yangyuhua@nuc.edu.cn (Y.Y.); wdzhang@nuc.edu.cn (W.Z.)

**Keywords:** MEMS, CMUT array, sensitivity, time of flight (ToF), thickness detection

## Abstract

This paper presents the design, fabrication, and experimental evaluation of a capacitive micromachined ultrasonic transducer (CMUT) linear array for non-contact thickness measurement of marine engineering structures. A 16-element CMUT array was fabricated using a silicon–silicon wafer bonding process, and encapsulated in polyurethane to ensure acoustic impedance matching and environmental protection in underwater conditions. The acoustic performance of the encapsulated CMUT was characterized using standard piezoelectric transducers as reference. The array achieved a transmitting sensitivity of 146.82 dB and a receiving sensitivity of −229.55 dB at 1 MHz. A complete thickness detection system was developed by integrating the CMUT array with a custom transceiver circuit and implementing a time-of-flight (ToF) measurement algorithm. To evaluate environmental robustness, systematic experiments were conducted under varying water temperatures and salinity levels. The results demonstrate that the absolute thickness measurement error remains within ±0.1 mm under all tested conditions, satisfying the accuracy requirements for marine structural health monitoring. The results validate the feasibility of CMUT-based systems for precise and stable thickness measurement in underwater environments, and support their application in non-destructive evaluation of marine infrastructure.

## 1. Introduction

In marine environments [1], ships [2], offshore engineering structures, and subsea pipelines are continuously subjected to factors such as seawater corrosion [3], pressure fluctuations [4], and biological fouling [5], where changes in material thickness can directly affect their safety, reliability, and service life. Accurate thickness measurement therefore represents a critical inspection technique for ensuring the structural integrity of marine assets. Compared with alternative methods such as X-ray [6], laser-based techniques, and mechanical contact testing, ultrasonic technology [7,8,9] has emerged as one of the most important non-destructive testing (NDT) solutions. This prominence is attributed to its distinct advantages: non-invasive operation, high measurement precision, adaptability to complex environments, compatibility with a wide range of materials, rapid testing capabilities, and the ability to penetrate opaque media. These features make ultrasonic methods indispensable for evaluating thickness variations in submerged or otherwise inaccessible structures [10,11,12].

Ultrasonic transducer arrays [13], serving as the core components for acoustic–electric signal conversion in ultrasonic thickness measurement systems, play a pivotal role in determining the overall performance of such systems. Compared with traditional piezoelectric transducers (e.g., Lead Zirconate Titanate (PZT)), microelectromechanical ultrasonic transducers (MUTs) exhibit significant advantages in underwater detection: First, MUTs reduce reliance on coupling agents through the thin-film vibration mechanism, with an acoustic impedance matching error of <5% within the 1–10 MHz frequency band [14]. Second, the influence of frequency on resolution is notable: MUTs can achieve an axial resolution of <300 μm at high frequencies (e.g., 10 MHz), which is superior to the >1 mm resolution of PZT at low frequencies (<5 MHz) [15]. Third, the integration capability with complementary metal-oxide semiconductors (CMOSs) enables the system power consumption to be reduced to 3.8 mW and the chip area to be controlled within 4 mm^2^, far outperforming the discrete integration scheme of PZT. In contrast, capacitive micromachined ultrasound transducers (CMUTs), a new generation of ultrasonic transducers based on microelectromechanical system (MEMS) technology, emerge as a promising alternative. The integration capability of MEMS technology is a defining advantage, enabling the fabrication of 2D arrays with thousands or even tens of thousands of micro-scale elements and facilitating monolithic integration with signal processing circuits [16]. This not only expands the detection coverage but also significantly enhances data acquisition precision. In addition, CMUTs offer superior features, such as wide bandwidth, excellent underwater adaptability eliminating the need for coupling agents, and low power consumption. As CMUT technology continues to advance, its potential for enabling high-performance, scalable, and environmentally robust thickness measurement systems makes it an indispensable solution for non-destructive evaluation in offshore engineering structures, poised to drive transformative advancements in marine asset integrity management [14,17,18,19,20].

Compared with single-element CMUT transducers, CMUT linear array transducers enable simultaneous waveform emission from multiple elements, leveraging signal energy superposition to enhance ultrasonic transmission intensity. This augmentation significantly improves penetration through thick or highly attenuating materials, ensuring robust reflection signal strength during reception and thereby enhancing measurement feasibility in challenging scenarios. Meanwhile, the array configuration allows for data averaging or fusion across multiple elements [21], capitalizing on the randomness of individual measurement errors to mitigate overall errors and improve measurement precision [22]. Additionally, the spatial distribution of array elements expands the effective detection area, enabling efficient coverage of large-scale structures or multi-point thickness measurements. This feature drastically reduces inspection time and operational workload compared to sequential single-element scanning. Given these combined advantages in signal strength, measurement accuracy, and detection efficiency, CMUT linear array transducers are selected in this study for thickness measurement applications.

In ultrasonic measurement techniques, the ToF method [23], as one of the earliest developed approaches, has been widely adopted across diverse ultrasonic measurement applications [24]. In 2016, Zhang et al. [25] demonstrated ultrasonic measurement of oil film thickness using piezoelectric transducers via the ToF principle. More recently, in 2022, B. Bühling et al. introduced a non-contact air-coupled ultrasonic ToF measurement method based on laser Doppler vibrometry, achieving a distance measurement error of less than 1.5% [26]. However, despite these advancements, the application of CMUT linear array transducers to construct thickness detection systems for offshore engineering structures using the ToF method remains unexplored in the current literature, representing an unaddressed research gap in ultrasonic non-destructive evaluation for marine environments.

Addressing the application requirements of ultrasonic transducers in offshore engineering structure thickness detection systems, this study designed and fabricated a high-density integrated CMUT linear array transducer, followed by comprehensive performance characterization of the as-prepared device. The developed detection system utilizes the CMUT linear array to emit and receive ultrasonic signals, determining object thickness via the ToF method by recording the time delay between reflected signals from the front and back interfaces. Environmental impacts on measurement accuracy—specifically temperature and salinity variations—were systematically evaluated to assess the system’s robustness in complex marine conditions. The thickness detection system constructed using CMUT transducers demonstrates substantial application potential for precise, reliable thickness measurement in offshore engineering structures, addressing the critical need for non-destructive evaluation in harsh marine environments.

## 2. CMUT Linear Array Design and Fabrication

### 2.1. Design

**Figure 1:**
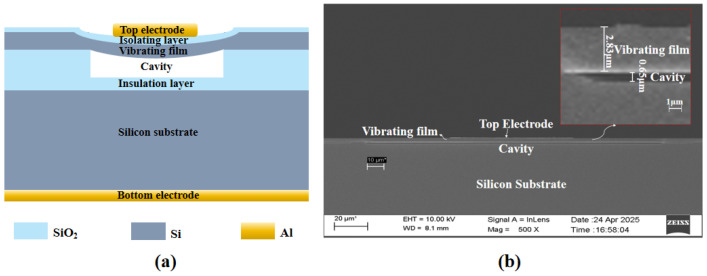
CMUT linear array transducers. (**a**) Working principle diagram of a sensing cell. (**b**) SEM image of the cross-section of the CMUT array (20 μm corresponds to the scale bar in the SEM image).

Figure 1a illustrates the CMUT cell structure, which consists of a movable top electrode diaphragm, a fixed lower electrode layer, and an insulating dielectric layer sandwiched between the two electrodes. The movable upper electrode diaphragm is constructed from a deformable single-crystal silicon membrane integrated with a patterned metal electrode. This diaphragm structure undergoes mechanical deformation and generates vibrations under voltage excitation for ultrasonic emission, or conversely, vibrates in response to incident acoustic pressure signals during reception, enabling bidirectional acoustic–electric conversion.

A vacuum cavity is formed between the movable single-crystal silicon membrane and the monocrystalline silicon substrate, with a silicon dioxide insulating layer retained at the bottom of the vacuum cavity. This design prevents the movable membrane from collapsing and making direct contact with the substrate, thereby avoiding high-voltage breakdown within the device due to electrode short-circuiting. The cross-sectional scanning electron microscope (SEM) image performed by the TESCAN MIRA3 of a sensing cell in the MEMS hydrophone sensor is shown in Figure 1b. Table 1 lists the structural parameters of the CMUT cell, where the thickness of the movable single-crystal silicon membrane is 2.83 μm and the vacuum gap height is 0.65 μm.

**Figure 2:**
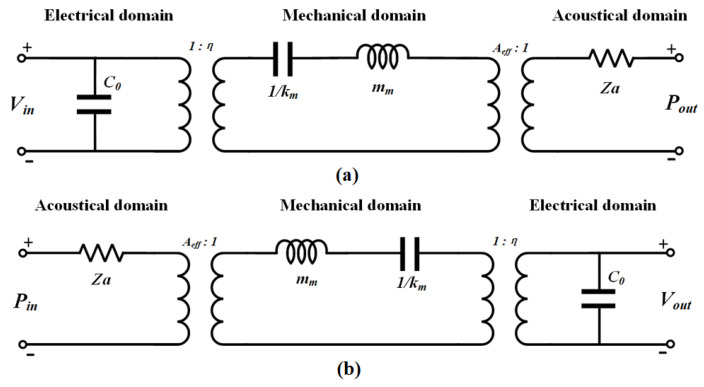
Equivalent circuit model of the CMUT sensing cell, which includes the acoustic, mechanical, and electrical domains. (**a**) Transmission equivalent circuit model. (**b**) Receiving equivalent circuit model.

During operation, CMUTs involve bidirectional conversion between electric field forces and mechanical vibrations, with mechanical motions coupled to ultrasonic waves during transmission or inducing mechanical responses from incident ultrasonic waves during reception. The equivalent circuit models for the CMUT ultrasonic transducer sensing cell [27] are depicted in Figure 2, where Figure 2a represents the model under transmission mode and Figure 2b illustrates the model under reception mode.

In transmission mode, an applied excitation voltage $V_{in}$ is imposed across the CMUT electrodes, generating electrostatic forces that induce vibration of the CMUT membrane and subsequently produce ultrasonic signals in the coupling medium. In the electrical domain, $C_{0}$ represents the static capacitance, and energy conversion from the electrical to mechanical domain is achieved via an ideal transformer with a turns ratio of 1:η. In the mechanical domain, the model incorporates the effective mass $m_{m}$ and effective stiffness $k_{m}$, which characterize the sensing cell dynamic behavior for a specific vibration mode. In the acoustical domain, $P_{out}$ denotes the output acoustic pressure, and $Z_{a}$ represents the acoustic impedance, which is coupled to the water through an area conversion ratio of $A_{eff}:1$. The overall input impedance in the frequency domain is expressed as:

(1) $$Z_{in}\left(s\right)=\frac{1}{sC_{0}}+\frac{1}{\eta^{2}}\left(sm_{m}+\frac{1}{sk_{m}}+\frac{Z_{a}}{A_{eff}^{2}}\right)$$

In reception mode, the CMUT surface is subjected to an external ultrasonic acoustic pressure $P_{in}$, which generates an equivalent driving force causing the membrane to undergo mechanical displacement. This displacement induces charge variations between the electrodes, thereby generating an output voltage $V_{out}$. In the acoustic domain, the incident acoustic pressure $P_{in}$ acting on the sensing cell effective area $A_{eff}$ is converted into a mechanical force $F=A_{eff}\cdot P_{in}$, which drives mechanical vibrations in the mechanical domain. $Z_{a}$ is the acoustic impedance. The mechanical displacement X(s) is converted into electrical charge through electromechanical transformation with a turns ratio η. In the electrical domain, $C_{0}$ denotes the static capacitance of the sensing cell, and $V_{out}$ represents the output voltage generated by the charge variation on the sensing surface. In the frequency domain, the relationship between the output voltage and the input acoustic pressure can be characterized by a transfer function.

(2) $$V_{out}\left(s\right)=\frac{\eta A_{eff}}{C_{0}\left(sm_{m}+\frac{1}{sk_{m}}+\frac{Z_{a}}{A_{eff}^{2}}\right)}P_{in}\left(s\right)$$

where *s* denotes the Laplace variable in the frequency domain.

### 2.2. Fabrication

When operating in a vacuum, the resonant frequency of a CMUT is denoted as $f_{0}$ [28]. In contrast, when the CMUT is immersed in a liquid environment with density $\rho_{L}$, its resonant frequency shifts to $f_{1}$ [29].

(3) $$f_{0}=\frac{0.467h}{a^{2}\sqrt{\frac{E}{\rho (1-\sigma^{2})}}}$$

(4) $$f_{1}=\frac{0.467h}{a^{2}\sqrt{\frac{E}{\rho \left(1-\sigma^{2}\right)}}}/\sqrt{1+0.67\rho_{L}a/\rho h}$$

where *h* is the thickness of the membrane, *a* is the radius of the membrane, *E* is the Young’s modulus of the membrane material, ρ is the material density, and σ is the Poisson’s ratio of the thin film. From the equation, it can be observed that the resonant frequency of the membrane is exclusively determined by its geometric dimensions and material properties. For a given membrane material, a larger radius or smaller thickness leads to a higher resonant frequency, reflecting the inverse relationship between frequency and membrane size under fixed material conditions [30].

To ensure sufficient detection depth for underwater operation, the CMUT in this study was designed to have a resonant frequency of 1 MHz. Previous studies have indicated that excessively thin membranes are prone to vibration fatigue, leading to structural damage, whereas overly thick membranes necessitate higher driving voltages to achieve effective actuation. Balancing these trade-offs, a membrane thickness of 2.83 μm was determined as the optimal compromise, ensuring both mechanical durability and operational efficiency under the target frequency.

**Figure 3:**
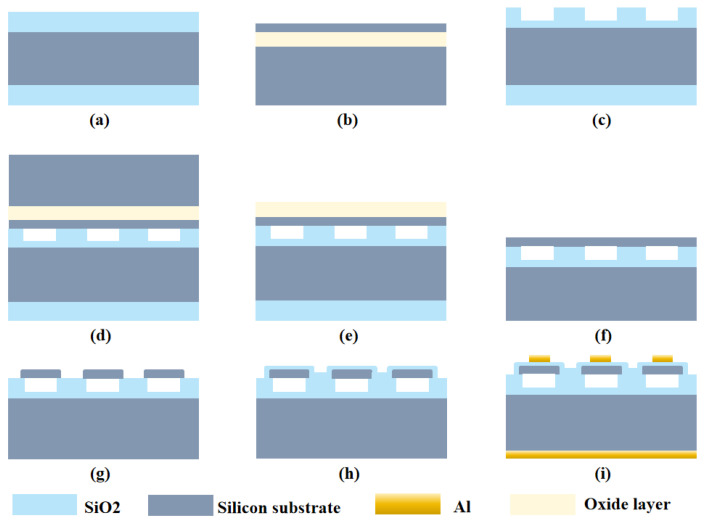
Processing flowchart of CMUT based on wafer bonding. (**a**) Thermal oxidation of the silicon wafer. (**b**) SOI wafer. (**c**) Etching of the cavity and alignment markers. (**d**) Wafer bonding of the SOI wafer. (**e**) Removal of silicon substrate. (**f**) Removal of the oxide layer. (**g**) Etching isolation grooves and marking grooves. (**h**) Chemical vapor deposition oxide layer. (**i**) Sputtering on the upper and lower electrodes.

This study presents a MEMS microfabrication process for CMUT linear array transducers, with silicon–silicon wafer bonding as the core technique to fabricate the CMUT array device [31]. The detailed fabrication process of the CMUT array is illustrated in Figure 3. An oxidized silicon wafer with an 0.8 μm thick thermal oxide layer and a silicon-on-insulator (SOI) wafer were first prepared. After photolithographically patterning and etching 0.65 μm deep cavities on the front side of the oxidized silicon wafer, it was pre-bonded with the SOI wafer at room temperature under vacuum conditions. It is critical to ensure sufficient vacuum level and surface cleanliness to prevent the formation of unbonded regions or micro-voids. The bonded wafers were then annealed in a high-temperature oxidation furnace, with uniform temperature distribution during annealing to avoid stress discrepancies caused by inconsistent bonding strength.

The substrate of the SOI wafer was thinned via chemical mechanical polishing (CMP), taking care to avoid membrane rupture. The remaining silicon substrate was etched away using a silicon etchant, followed by removal of the buried oxide layer of the SOI wafer with buffered oxide etchant (BOE) to form a free-standing silicon membrane structure. Silicon etching and silicon dioxide (SiO_2_) deposition were subsequently performed; during etching, over-etching (which would cause membrane thickness to exceed the designed value) and under-etching (which would leave residual substrate) must be avoided to prevent yield degradation.

A metal layer was sputtered onto the SiO_2_ layer and patterned to form the top electrode of the capacitor. Precise alignment during patterning is essential to prevent short circuits between the top and bottom electrodes. On the substrate side, a 1 μm thick metal layer was deposited and annealed to achieve ohmic contact between the substrate and metal, serving as the bottom electrode of the capacitor.

**Figure 4:**
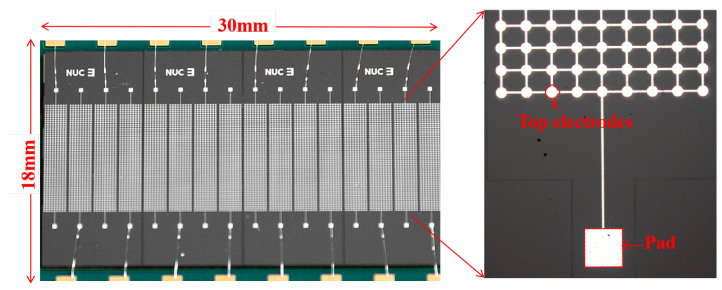
Optical microscope image of the CMUT manufactured in this work.

Figure 4 presents a top-view image of the CMUT array, where the CMUT linear array transducer is composed of 16 elements. Each element is configured with 90 × 9 (totaling 810) micro-capacitive units arranged in parallel, forming a high-density integrated structure to enhance acoustic signal sensitivity and array uniformity.

## 3. Development and Operational Principle of Thickness Detection System

To prevent direct contact between CMUT chip surfaces and water—as such contact could induce electrical short circuits or corrosion of microstructures—CMUT arrays require packaging. In non-contact ultrasonic thickness measurement using water as the coupling medium, the encapsulation layer material of the transducer plays a decisive role in enabling effective acoustic energy coupling and propagation. The encapsulation material must not only exhibit high acoustic transparency but also possess acoustic impedance characteristics matched to water, the coupling medium, to minimize interfacial reflection and enhance measurement sensitivity and resolution. This dual requirement ensures minimal energy loss at the transducer–medium interface, thereby optimizing the signal-to-noise ratio and enabling precise thickness detection in non-destructive evaluation applications [32]. Acoustic impedance is determined by the material density ρ and the longitudinal wave velocity ν and can be expressed as follows:

(5) Z=ρν

The acoustic impedance of water is approximately $1.48\times 10^{6}\left(\mathrm{kg}/m^{2}\cdot s\right)$, while that of commonly used polyurethane materials ranges from 1.2 to $1.7(\mathrm{kg}/m^{2}s)$, depending on their curing density, degree of molecular crosslinking, and additive composition. This range closely matches the acoustic impedance of water, thereby creating a low reflectivity at the water–polyurethane interface. This matching minimizes energy loss due to reflection, enabling efficient transmission of ultrasonic energy into the target structure and back to the transducer, which is critical for maintaining high-fidelity signal transduction in non-contact thickness measurement systems.

According to the reflection coefficient formula

(6) $$R={\left(\frac{Z_{2}-Z_{1}}{Z_{2}+Z_{1}}\right)}^{2},$$

when the acoustic impedance of polyurethane, $Z_{2}$, closely matches that of water, $Z_{1}$, the reflection coefficient R approaches zero. This indicates minimal energy reflection at the interface, allowing the majority of ultrasonic waves to propagate through unimpeded. Such impedance matching is critical in non-contact ultrasonic thickness measurement—especially in dual-interface systems involving water, the encapsulation layer, and the target material—as it significantly enhances the quality of echo signals and the system’s signal-to-noise ratio (SNR). Beyond impedance matching, polyurethane exhibits additional advantageous properties: it combines excellent flexibility with mechanical strength [33,34], providing robust physical protection for the ultrasonic transducer. Furthermore, its low water absorption and hydrolysis resistance ensure stable performance during long-term underwater operation [35,36], minimizing performance drift. Given these attributes, the 16-element CMUT linear array developed in this study was encapsulated with polyurethane, rendering it well-suited for non-contact ultrasonic thickness measurement in aquatic environments.

**Figure 5:**
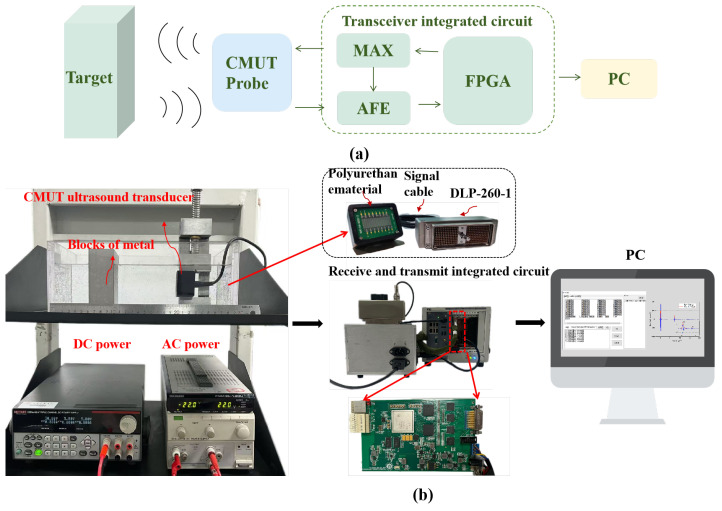
Schematic diagram of the CMUT-based thickness detection system. (**a**) Functional block. (**b**) Testing process.

**Figure 6:**
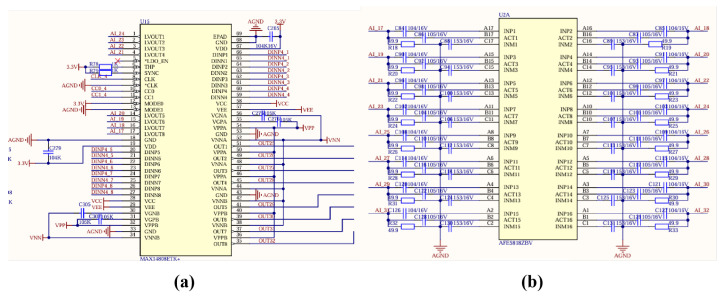
Schematic diagrams of the transmit–receive integrated circuit. (**a**) Schematic diagram of the transmit circuit. (**b**) Schematic diagram of the receive circuit.

A thickness measurement system for marine engineering structures was developed using a 16-element CMUT linear array transducer, with its conceptual diagram illustrated in Figure 5a. The experimental test platform is shown in Figure 5b. The target specimen and the 16-element linear array transducer were vertically positioned and rigidly fixed within a water tank, maintaining a 10 cm acoustic path between them. The transducer was connected to a transmit–receive integrated circuit, where a 30V DC bias voltage and a 22V amplitude AC excitation signal were applied to its electrodes to generate ultrasonic waves. A transmit–receive integrated circuit is used to drive the CMUT for transmitting and receiving waveforms, as shown in Figure 6a. The MAX14808 high-voltage pulse excitation chip is employed as the core component of the transmit circuit to drive the CMUT transducer, as illustrated in Figure 6b. The ultrasonic receiving circuit is constructed using the AFE5818 chip. When detecting CMUT echo signals, the AFE chip is configured as follows: the echo signal passes through the TR switch of the MAX chip, flows out from the LVOUT pin, and enters the input pins INP and INM of the AFE chip. Upon transmission, the received echo signals were sent to a PC for real-time waveform display, enabling systematic characterization of the transducer’s performance in water-based non-destructive thickness measurement.

The core of this system relies on the ToF method to estimate the thickness of the target object, combined with high-resolution signal processing techniques to enhance time delay extraction accuracy. This process involves recording the echo signals reflected from the target interfaces and extracting the time delay between the primary peaks of the front and back surface waveforms—denoted as ToF. Assuming the sound velocity within the specimen is ν, the thickness *d* can be calculated using the following equation:

(7) $$d=\frac{\nu *ToF}{2}$$

The division by 2 in the equation accounts for the round-trip travel of the ultrasonic wave through the specimen. In this study, echo signals acquired from the 16 array elements were first averaged to reduce noise, followed by envelope processing using an absolute-value low-pass filtering method to highlight the signal amplitude variations. A quadratic parabola interpolation technique was then employed to achieve sub-sample level correction for the envelope peak positions, enabling a time precision of 0.01 μs in ToF extraction. The specific interpolation procedure is as follows.

(8) $$\delta =\frac{1}{2}\cdot \frac{y_{1}-y_{3}}{y_{1}-2y_{2}+y_{3}}$$

(9) $$t_{\mathrm{fine}}=\left(i+\delta \right)\cdot \Delta t$$

where, $y_{1}$, $y_{2}$, and $y_{3}$ represent three consecutive sampled values around the envelope peak, $y_{2}$, at the peak position, $\Delta_{t}$ denotes the sampling interval (0.1 μs), and *i* represents the integer sampling point index adjacent to the peak point of the envelope signal, which is an integer corresponding to the position of the original sampling point. δ represents the correction.

**Figure 7:**
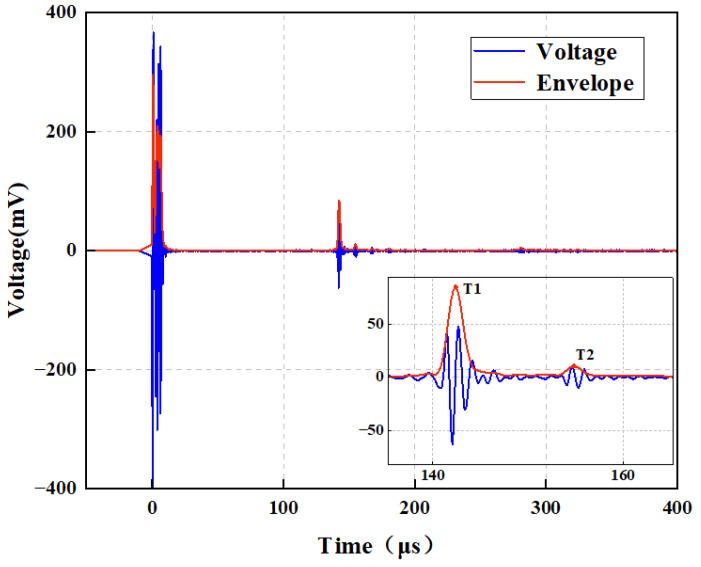
Transmitted and received signals collected by the system.

The processed waveform is shown in Figure 7, where $T_{1}$ and $T_{2}$ indicate the primary peak positions of the front and back surface reflection signals from the specimen, respectively. These peaks are identified after envelope processing and sub-sample interpolation, providing precise time markers for calculating the ToF with sub-microsecond accuracy.

In the marine engineering field, structures such as ship hulls, seawater pipelines, offshore platforms, and wind turbine substructures are subjected to harsh environments characterized by high salinity, humidity, and wave impacts during long-term service. The selected metallic materials must exhibit excellent corrosion resistance, a balanced strength–toughness ratio, and good weldability. Carbon steel, valued for its low cost, reliable mechanical properties, and ease of welding, is widely used in the main structures of offshore platforms and ship hulls. Stainless steel, particularly 316 L, offers superior resistance to pitting, crevice corrosion, and stress corrosion cracking, making it ideal for pipeline systems and critical connection components in direct seawater contact [37]. Aluminum alloys, prized for their high strength-to-weight ratio, are essential in weight-sensitive marine structures such as high-speed craft, boarding platforms, and buoys.

To validate the CMUT-based thickness measurement system, three representative materials were selected as test specimens: 6061 aluminum alloy, 316 L stainless steel, and AH36 carbon steel, with thicknesses of 20 mm, 40 mm, 60 mm, and 80 mm. Prior to thickness measurement, sound velocity calibration for each material was performed using the pulse-echo method in accordance with the international standard GB/T 23900-2009 [38], ensuring measurement accuracy through systematic characterization of acoustic propagation properties. The calibration process involved transmitting ultrasonic pulses through the specimens and recording round-trip travel times, from which material-specific sound velocities were derived to enable precise thickness calculations. Table 2 shows the acoustic velocities of the calibration blocks: the acoustic velocity of 6061 aluminum alloy is 6299.2 m/s, that of 316L stainless steel is 5743 m/s, and that of AH36 carbon steel is 5873.1 m/s.

## 4. Experimental Results and Discussion

**Figure 8:**
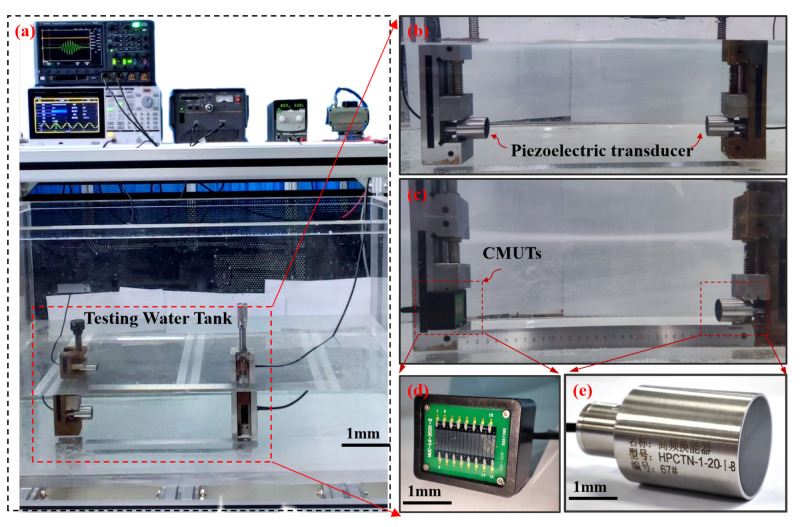
Experimental testing platform. (**a**) Testing process. (**b**) Calibration of piezoelectric transducer under receiving sensitivity test. (**c**) Transmit voltage response test. (**d**) Polyurethane material. (**e**) Standard 1 MHz piezoelectric transducer.

**Figure 9:**
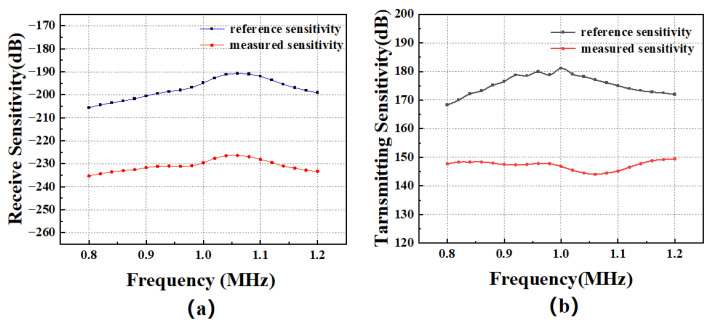
The performance parameters. (**a**) Transmitting sensitivity curve of the CMUT linear array transducer and standard transducer. (**b**) Receiving sensitivity curve of the CMUT linear array transducer and standard transducer.

The transmitting sensitivity and receiving sensitivity are critical parameters for evaluating the transmission and reception performance of CMUT linear array transducers. A dedicated test platform was constructed, as shown in Figure 8, to characterize these metrics. The transmitting sensitivity of the CMUT array was measured in a water tank at a distance of approximately 30 cm across a frequency range of 0.8MHz–1.2MHz. As shown in Figure 9a, the reflection response spectrum and final emission response spectrum of the standard transducer show a transmit voltage response (TVR) of 146.82dBat1MHz, indicating effective generation of sound waves under the specified excitation conditions. For receiving sensitivity characterization, a reciprocal calibration method was employed using a standard 1 MHz piezoelectric transducer as the reference. This approach involved measuring the electrical output of the CMUT element in response to known acoustic pressures across the same frequency range. As shown in Figure 9b, the receiving sensitivity of the standard transducer and the tested CMUT is determined to be −229.55dBat1MHz, demonstrating the transducer’s ability to convert incident acoustic energy into high fidelity electrical signals. These measurements validate the CMUT array’s suitability for high-precision thickness detection in marine environments, where both robust signal transmission and sensitive reception are essential for reliable non-destructive evaluation.

**Figure 10:**
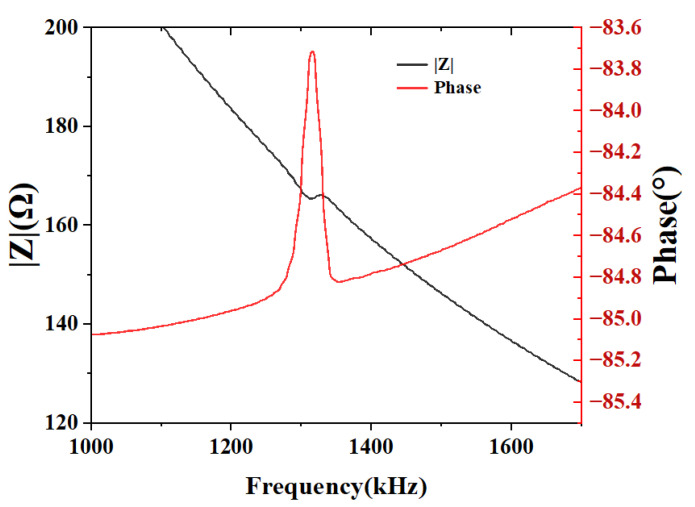
Impedance curve of the CMUT array in air.

The impedance of the CMUT array measured in air by an impedance analyzer (Keysight E499oA) is shown in Figure 10. As can be seen from the impedance frequency spectrum, the resonant frequency in the air for the CMUT array is 1311 kHz and the anti-resonant frequency is 1330 kHz. As can be seen from the figure, the phase curve shows an overall increasing trend, but its maximum value remains negative, indicating that the capacitive property of the CMUT dominates. The Z curve exhibits an overall decreasing trend, suggesting that the capacitive reactance decreases with increasing frequency.

To analyze the impact of environmental factors in marine environments on the accuracy of CMUT-based thickness measurement, thickness measurements were carried out over a temperature range of 10 °C–30 °C (in increments of 5 °C) and a salinity range of practical salinity units of 0.1–0.35% (in increments of 0.05%). This experimental design systematically evaluates how temperature and salinity variations affect measurement precision, critical for ensuring reliable non-destructive testing in harsh offshore conditions where water properties can fluctuate significantly.

**Figure 11:**
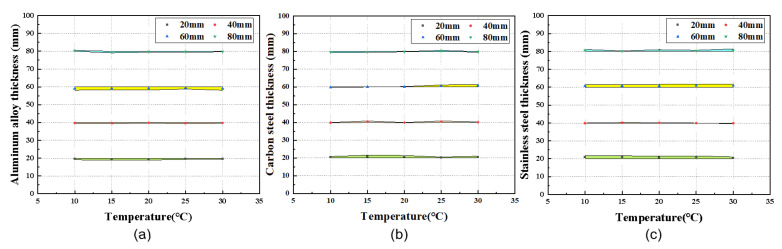
Measured data of specimens with different thicknesses at different temperatures. (**a**) Aluminum alloy. (**b**) Carbon steel. (**c**) Stainless steel.

To investigate the influence of temperature variations on the thickness measurement accuracy of the CMUT system, the following experimental protocol was implemented: A sealed container filled with pure water was first cooled to 5 °C using a DHC-05 low-temperature constant-temperature water bath. The cooled water was then transferred to the test water tank containing the CMUT linear array transducer. A MJ-R10 constant-temperature heater was used to incrementally raise the water temperature in 5 °C steps up to 30 °C. At each target temperature, the water was stabilized within ±0.2 °C of the setpoint for 3 min before measurements were initiated. Water temperature was continuously monitored in real-time using a high-precision thermocouple thermometer (Type k; measurement accuracy: $\pm 0.1^{\circ }C$). Each temperature condition was tested five times, with the average value used for the analysis. Figure 11 illustrates the relationship between specimen thickness and temperature variations across different materials. The results demonstrate that, over the 10 °C–30 °C temperature range, thickness measurement precision remained within ±0.1mm, and relative errors were confined to within ±0.5% of the target thickness. These findings confirm that temperature fluctuations do not significantly impact the measurement accuracy of the thickness detection system, satisfying the error tolerance requirements for marine engineering structural thickness testing.

In the experiment investigating the effect of salinity on thickness measurement accuracy, salt solutions with mass fractions of 0%, 0.1%, 0.15%, 0.20%, 0.25%, 0.30%, and 0.35% were prepared by dissolving pure NaCl in deionized water according to the following formula. This method ensures precise control over the salinity gradient, with each solution meticulously formulated to achieve the target concentration by systematically varying the mass of NaCl added while maintaining a constant total solution volume. The use of deionized water as the base medium minimizes impurities that could interfere with acoustic propagation, allowing for isolated evaluation of salinity effects on measurement performance.

(10) $$m_{l}=s*\frac{m_{h}}{1000-s}$$

**Figure 12:**
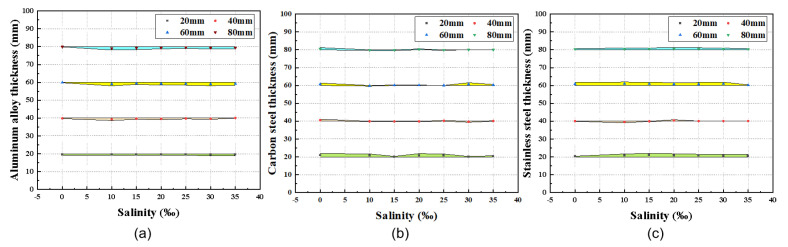
Measured data of specimens with different thicknesses at different salinity. (**a**) Aluminum alloy. (**b**) Carbon steel. (**c**) Stainless steel.

In this equation, $m_{l}$ represents the mass of solute required for preparation, *s* denotes the salinity concentration of the solution, and $m_{h}$ is the mass of water in the salt solution. For each target concentration, the required solute was weighed using an electronic balance, dissolved in deionized water through thorough stirring to ensure homogeneity, and calibrated using a salinity meter (measurement accuracy: ±0.1%). Under each salinity condition, specimens of different materials and thicknesses were fully immersed in the salt solution, with measurements repeated five times per specimen to calculate average values for analysis. Figure 12. depicts the relationship between specimen thickness and salinity variations across different materials. The results show that in pure water, the thickness measurement precision of the system remained within ±0.1mm. As the salinity concentration was varied, the absolute error band stayed within ±0.1 mm relative to the standard thickness, with relative errors confined to ±0.5% of the measured thickness. These error margins fall within the controllable range, confirming that salinity fluctuations do not significantly affect the measurement accuracy of the thickness detection system. This performance meets the error tolerance requirements for thickness testing of marine engineering structures, validating the system’s reliability in saline environments. Overall, the marine engineering structural thickness detection system developed using a 16-element CMUT linear array transducer with the ToF method exhibits measurement accuracy unaffected by environmental factors such as temperature and salinity, demonstrating deterministic and stable performance across the tested conditions.

## 5. Conclusions

In this work, a 16-element CMUT linear array was designed, fabricated, and characterized for non-contact ultrasonic thickness measurement in marine engineering structures. It is encapsulated with polyurethane for underwater environments to reduce the acoustic impedance mismatch rate between the CMUT and water, thereby significantly reducing signal loss. The transmit voltage response level is 146.82 dB@1 MHz, and the receive sensitivity is −229.55 dB@1 MHz [39]. When integrated with a custom transmit–receive circuit, the system enhances the signal-to-noise ratio (SNR) and receive gain [40], and employs the time-of-flight (ToF) method to perform accurate thickness measurements without requiring physical contact. The influence of environmental parameters, specifically water temperature (10 °C–30 °C) and salinity (0–0.35%), was experimentally evaluated. The results showed that the system maintained a measurement accuracy within ±0.1mm in all conditions tested, meeting the practical requirements for offshore structural monitoring. These findings validate the robustness and reliability of the CMUT array in variable marine environments and highlight its potential for scalable, high-precision, and non-invasive thickness evaluation in demanding offshore applications.

## Figures and Tables

**Table 1:** Key parameters for CMUT array.

Parameters	Value (μm)
Monocrystalline silicon thickness	2.83
Cavity diameter	180
Cavity height	0.65
Cavity insulation thickness	0.15
Oxide thickness	1

**Table 2:** Sound velocity of calibration block.

Serial No.	Material of Calibration Block(m/s)	Sound Velocity
1	6061 aluminum alloy	6299.2
2	316 L stainless steel	5743.0
3	AH36 carbon steel	5873.1

## Data Availability

All data supporting this study are included in the article.
